# Association of Serum ADA Levels in Pulmonary Tuberculosis: A Systematic Review and Meta-Analysis

**DOI:** 10.3390/ijerph23040498

**Published:** 2026-04-14

**Authors:** Jirarat Songsri, Jongkonnee Thanasai, Jitbanjong Tangpong, Anchalee Chittamma, Wiyada Kwanhian Klangbud

**Affiliations:** 1 School of Allied Health Sciences, Walailak University, Nakhon Si Thammarat 80160, Thailand; jirarat.so@wu.ac.th (J.S.); rjitbanj@wu.ac.th (J.T.); 2 Center of Excellence Research for Melioidosis and Microorganisms (CERMM), Walailak University, Thasala, Nakhon Si Thammarat 80160, Thailand; 3 Faculty of Medicine, Mahasarakham University, Mahasarakham 44000, Thailand; jongkonnee@msu.ac.th; 4 Department of Pathology, Faculty of Medicine Ramathibodi Hospital, Mahidol University, Bangkok 10400, Thailand; anchalee.chi@mahidol.ac.th; 5 Medical Technology Program, Faculty of Science, Nakhon Phanom University, Nakhon Phanom 48000, Thailand

**Keywords:** pulmonary tuberculosis, adenosine deaminase, ADA, serum, *Mycobacterium tuberculosis*

## Abstract

**Highlights:**

**Public health relevance—How does this work relate to a public health issue?**

- Pulmonary tuberculosis (PTB) remains a major global health challenge, with ongoing need for improved understanding of biomarkers associated with disease processes.
- This study evaluates the association between serum adenosine deaminase (ADA) levels and PTB as a marker of immune activation.

**Public health significance—Why is this work of significance to public health?**

- A meta-analysis of 34 studies demonstrates that serum ADA levels are significantly elevated in PTB patients compared with healthy individuals and those with other respiratory diseases.
- This association is consistently observed across diverse geographical regions and laboratory measurement methods, indicating a robust biological pattern.

**Public health implications—What are the key implications or messages for practitioners, policy makers and/or researchers in public health?**

- Elevated serum ADA reflects a consistent biological response to PTB, supporting its role as an immunological marker rather than a confirmed diagnostic tool.
- Further well-designed diagnostic accuracy studies are required before serum ADA can be considered for clinical or public health application.

**Abstract:**

**Background:** The early diagnosis of pulmonary tuberculosis (PTB) remains a global challenge. While serum adenosine deaminase (ADA) has been associated with tuberculosis-related immune activation, its consistency across different regions and laboratory methods remains unclear. This study aims to evaluate group-level differences in serum ADA levels and identify factors influencing these variations. **Methods:** A systematic search was conducted across PubMed, Embase, and Scopus up to February 2026. A meta-analysis using a random-effects model was performed to calculate the pooled standardized mean difference (SMD), reflecting group-level differences in serum ADA levels between PTB patients and control groups. **Results:** Thirty-four studies were included. Serum ADA levels were significantly higher in PTB patients compared to healthy controls (SMD = 3.15, 95% CI: [2.51–3.79], *p* < 0.0001) and other respiratory diseases (SMD = 2.06, 95% CI: [1.38–2.74], *p* < 0.0001). Subgroup analyses revealed that geographical region and ADA measurement methods did not significantly account for the observed high heterogeneity (*I*^2^ > 95%), indicating that ADA elevation was consistently observed across studies. **Conclusions:** Serum ADA levels were significantly elevated in patients with PTB, indicating a consistent biological association with disease status. However, given the high heterogeneity and the absence of diagnostic accuracy measures (e.g., sensitivity and specificity), these findings should not be interpreted as evidence of clinical diagnostic performance. Further diagnostic test accuracy studies are required to establish its clinical utility.

## 1. Introduction

Tuberculosis (TB) remains a major global public health challenge and continues to be one of the leading causes of morbidity and mortality worldwide. The disease, caused by *Mycobacterium tuberculosis*, primarily affects the lungs and manifests as pulmonary tuberculosis (PTB), the most common and transmissible form of the infection. Despite significant progress in TB control programs, early and accurate diagnosis remains difficult, particularly in resource-limited settings where the disease burden is highest [1]. Conventional diagnostic methods such as mycobacterial culture, although considered the gold standard, require several weeks to yield results due to the slow growth of M. tuberculosis. Rapid diagnostic techniques such as sputum smear microscopy are widely used but exhibit relatively low sensitivity, particularly in smear-negative or paucibacillary cases. While molecular diagnostic tools such as nucleic acid amplification tests have improved diagnostic sensitivity, their implementation is often restricted by high costs and the need for specialized laboratory infrastructure. Consequently, there is a growing demand for simple, rapid, and cost-effective biomarkers associated with disease-related immune activation; however, their clinical applicability remains to be established through diagnostic accuracy studies [1,2].

Adenosine deaminase (ADA), an enzyme involved in purine metabolism, has emerged as a biologically relevant marker associated with tuberculosis infection. ADA plays an important role in cellular immunity by regulating the proliferation and differentiation of T-lymphocytes, which are essential components of the host immune response against M. tuberculosis. Increased ADA activity has been observed in several forms of tuberculosis, reflecting the activation of cell-mediated immune mechanisms during infection [3]. Numerous studies have investigated ADA levels in various body fluids and have demonstrated elevated ADA levels associated with tuberculosis in specific clinical contexts, particularly in extrapulmonary tuberculosis. For instance, a meta-analysis by Goto et al. (2003) reported a high sensitivity and specificity of ADA in pleural fluid for diagnosing tuberculous pleural effusion, supporting its biological relevance in tuberculosis-related immune responses [4]. Similarly, Zhou et al. (2022) demonstrated the strong diagnostic performance of ADA in abdominal tuberculosis, further highlighting the enzyme’s role as a biologically relevant marker in TB-related inflammatory responses [5].

In addition to body fluid analysis, several clinical studies have evaluated serum ADA as a non-invasive biomarker associated with pulmonary tuberculosis. For example, Ali et al. (2024) reported elevated serum ADA levels in patients with smear-negative pulmonary tuberculosis compared with non-tuberculosis controls, suggesting that serum ADA is associated with disease status [6]. Moreover, recent research by Arghir et al. (2025) demonstrated that serum ADA levels are associated with inflammatory activity and disease severity in pulmonary tuberculosis patients [7]. These findings indicate that serum ADA has been reported as a biomarker associated with pulmonary tuberculosis and disease-related immune activity.

However, despite these encouraging findings, the association of serum ADA in pulmonary tuberculosis remains controversial. While several studies report significantly elevated ADA levels in PTB patients, others have found overlapping values between tuberculosis and other inflammatory or respiratory diseases, which may reduce its specificity. Furthermore, most existing research has focused primarily on ADA levels in extrapulmonary tuberculosis or specific body fluids, such as pleural, cerebrospinal, or ascitic fluid, rather than systematically evaluating serum ADA in pulmonary disease. For example, the meta-analysis conducted by Goto et al. (2003) primarily examined ADA in pleural effusions [4], while the systematic review by Pormohammad et al. (2017) focused on cerebrospinal fluid ADA in tuberculous meningitis. Although these studies demonstrate the biological relevance of ADA in tuberculosis, their findings cannot be directly generalized to pulmonary tuberculosis diagnosed using serum biomarkers [3]. Additionally, the study by Arghir et al. (2025) primarily explored the relationship between ADA and disease severity rather than evaluating its overall diagnostic performance [7]. Consequently, there remains a lack of comprehensive quantitative evaluation of the association of serum ADA with pulmonary tuberculosis.

To address these limitations, the present study conducts a systematic review and meta-analysis to evaluate group-level differences in serum adenosine deaminase levels in pulmonary tuberculosis. By synthesizing evidence from multiple clinical studies across diverse populations, this study aims to assess the biological association of serum ADA with PTB and to explore potential sources of heterogeneity among studies. Through subgroup analysis and meta-regression, this study further investigates methodological and regional factors influencing observed effect sizes.

## 2. Materials and Methods

### 2.1. Protocol Registration

This systematic review and meta-analysis was conducted in strict accordance with the PRISMA (Preferred Reporting Items for Systematic Reviews and Meta-Analyses) guidelines [8] (Supplementary Table S1). The study protocol was prospectively registered with PROSPERO (International Prospective Register of Systematic Reviews) under the registration number CRD420261324421.

### 2.2. Search Strategy

A comprehensive and systematic literature search was performed across three major electronic databases: PubMed, Scopus, and Embase, from their inception until 26 February 2026. The search strategy utilized a combination of Medical Subject Headings (MeSH) and free-text keywords related to the target population (e.g., “Tuberculosis, Pulmonary”, “*Mycobacterium tuberculosis*”), the biomarker of interest (“Adenosine Deaminase”, “ADA”), and the specimen type (“Serum”, “Blood”). Boolean operators (AND/OR) were employed to optimize search sensitivity and specificity. Strategy details are shown in Supplementary Table S2. The search was restricted to peer-reviewed original articles. Furthermore, the reference lists of all included studies and relevant review articles were manually screened to identify any additional eligible publications.

### 2.3. Eligibility Criteria

Studies were included if they met the following PICO criteria. Population (P) is patients diagnosed with pulmonary tuberculosis (PTB) confirmed by standard diagnostic methods. Intervention/Exposure (I) is measurement of serum ADA levels. (C) Comparison (C) is controlling groups comprising either healthy individuals or patients with non-TB respiratory diseases. Outcome (O) is sufficient quantitative data (mean and standard deviation) to calculate the standardized mean difference (SMD).

Exclusion criteria included: (1) studies focusing on pleural, meningeal, or extrapulmonary TB; (2) studies without a control group; (3) non-original research such as reviews, editorials, case reports, and conference abstracts; and (4) studies with insufficient data for effect size estimation despite attempts to contact the authors.

### 2.4. Data Extraction

Two independent reviewers extracted data using a standardized data collection form. Discrepancies were resolved through consensus or by consultation with a third senior investigator. The extracted information included: study characteristics (author, year of publication, country/region), participant demographics, sample sizes for both PTB and control groups, and the primary outcome data (mean and SD of serum ADA). For studies reporting data in medians and ranges/interquartile ranges, established statistical methods were used to estimate the mean and SD. For studies reporting medians and interquartile ranges or ranges, the mean and standard deviation were estimated using the method described by Wan et al. (2014), a widely used approach for estimating mean and standard deviation from median-based summary statistics [9].

### 2.5. Quality Assessment

The methodological quality of the included studies was independently assessed by two reviewers using the Newcastle–Ottawa Scale (NOS). The NOS evaluates three primary domains: the selection of study groups, comparability of the groups, and ascertainment of the exposure/outcome. Studies were categorized as high, moderate, or low quality based on their scores. Any disagreements in quality scoring were settled through discussion [10].

### 2.6. Statistical Analysis

The meta-analysis was performed using R software (version 4.4.2, R Foundation for Statistical Computing, Vienna, Austria) with the *meta* and *metafor* packages. The standardized mean difference (SMD) was utilized as the primary effect size to account for variations in ADA measurement units and laboratory techniques across studies. A random-effects model was applied for all analyses to provide more conservative estimates, assuming both within-study and between-study variability. These results indicate substantial differences in serum ADA levels between groups; however, SMD reflects the magnitude of separation rather than diagnostic discrimination.

Heterogeneity was assessed using the Cochran’s Q test and quantified using the *I*^2^ statistic, where *I*^2^ > 75 indicated high heterogeneity [11]. To explore potential sources of heterogeneity, subgroup analyses based on geographical regions were conducted, and meta-regression was performed using a mixed-effects model. Publication bias was evaluated through visual inspection of funnel plots and statistically confirmed by Egger’s linear regression test [12]. A *p*-value of <0.05 was considered statistically significant for all tests.

To assess the potential impact of non-normal data distributions, a sensitivity analysis was conducted excluding studies with a standard deviation greater than or approximately equal to the mean (SD/mean ≥ 1), which may indicate skewed distributions.

## 3. Results

### 3.1. Study Selection and Characteristics

**Figure 1:**
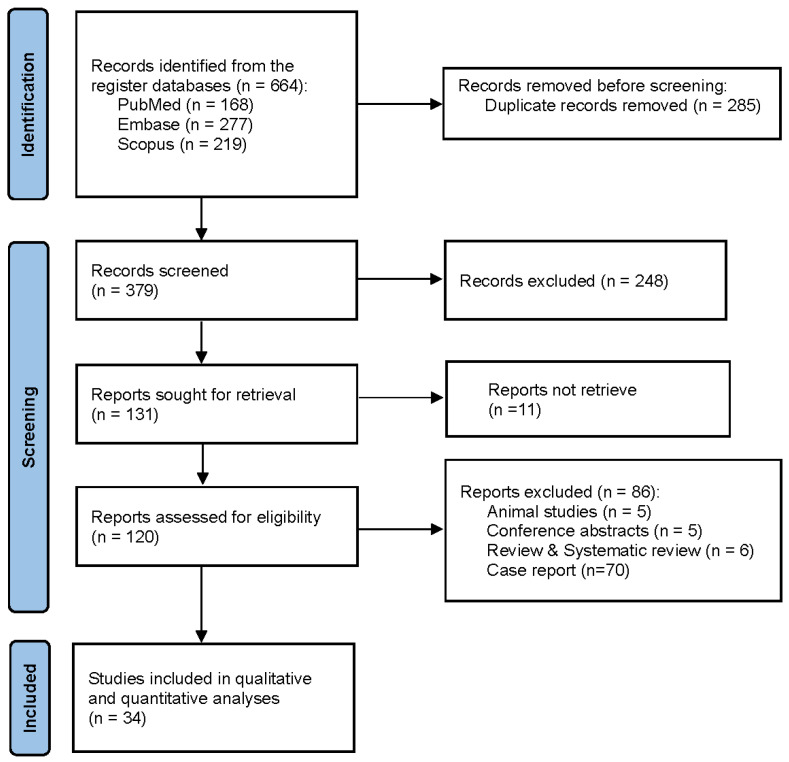
PRISMA flow diagram of study selection.

The systematic database search across PubMed, Scopus, and Embase yielded an initial total of 664 records. After removing duplicates and conducting a rigorous two-stage screening process—first by evaluating titles and abstracts, followed by a detailed full-text review—34 original studies met all eligibility criteria and were included in the final quantitative synthesis. The comprehensive step-by-step selection process, including the specific reasons for study exclusion at each stage, is visually detailed in the PRISMA flow diagram (Figure 1).

The 34 included studies [13,14,15,16,17,18,19,20,21,22,23,24,25,26,27,28,29,30,31,32,33,34,35,36,37,38,39,40,41,42,43,44,45,46] represent a broad geographical spectrum, encompassing research from South Asia, East Asia, the Middle East, Africa, and Europe. This diversity provides a robust global perspective on the observed differences in serum ADA levels in pulmonary tuberculosis (PTB). The fundamental characteristics of these studies—including the lead author, publication year, study location, sample size, and the specific ADA measurement techniques employed—are systematically summarized in Table 1.

Notably, the majority of the selected studies utilized either the traditional Giusti-based colorimetric assay or modern automated biochemistry analyzers. The predominant study designs were case–control and prospective cohorts. These foundational data points were further utilized to categorize the studies for subsequent subgroup analyses, aimed at investigating whether geographical location or laboratory methodology influenced the overall effect size and heterogeneity of the results.

### 3.2. Quality of Included Studies

The methodological quality of the 34 included studies was assessed using the Newcastle–Ottawa Scale (NOS), with a focus on selection, comparability, and exposure/outcome ascertainment (Supplementary Table S3). Overall, the included studies demonstrated an acceptable methodological quality. Twenty studies (58.8%) were classified as high quality (7–9 stars), characterized by definitive PTB diagnosis via sputum culture, smear microscopy, or nucleic acid amplification tests (NAATs), and rigorous demographic matching. Twelve studies (35.3%) were of moderate quality, primarily due to insufficient comparability or significant age discrepancies between cohorts (e.g., Abdelsadek et al., 2016 [13]; Afrasiabian et al., 2013 [14]). Two studies (5.9%) were deemed low quality due to selection bias and a limited sample size.

Exposure ascertainment was highly reliable, with most studies employing standardized laboratory techniques, notably the Giusti and Galanti method or automated kinetic colorimetric assays. This high prevalence of methodologically sound studies supports the internal validity of the pooled analysis of the pooled effect sizes in this meta-analysis.

### 3.3. Meta-Analysis of Serum ADA Levels in PTB Compared to Healthy Controls

#### 3.3.1. Pooled Standardized Mean Difference (SMD) in PTB vs Healthy

**Figure 2:**
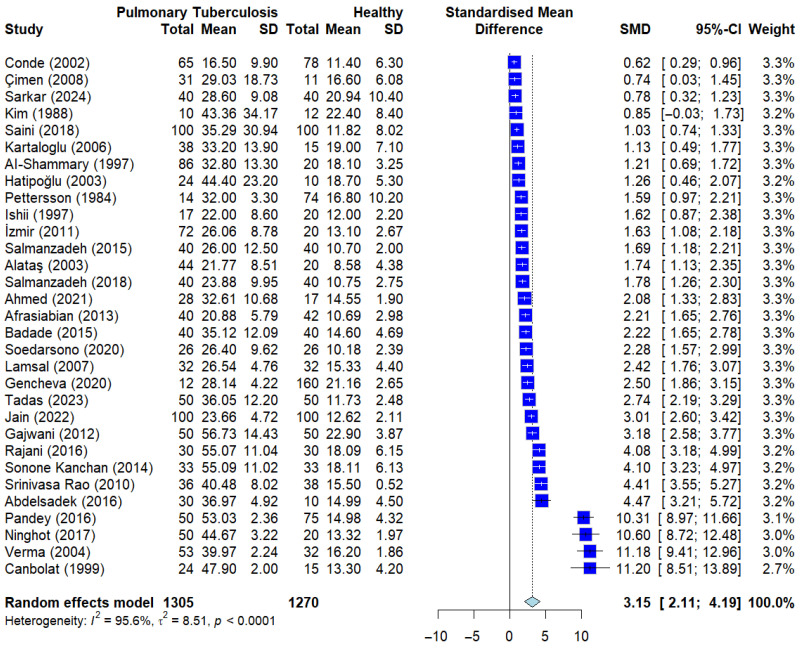
Forest plot of serum adenosine deaminase (sADA) levels in pulmonary tuberculosis b(PTB) patients vs. healthy controls. Deep blue squares represent individual study effect sizes, with horizontal lines indicating 95% confidence intervals. The size of each square reflects the study weight, and the blue diamond represents the pooled estimate from the random-effects model [13,14,15,16,17,20,21,22,23,25,26,27,28,29,30,31,32,33,34,35,36,37,38,39,40,41,42,43,44,45,46].

The primary meta-analysis, encompassing 31 independent studies, demonstrated a statistically significant elevation in serum adenosine deaminase (ADA) levels among patients diagnosed with pulmonary tuberculosis (PTB) compared to the healthy control cohorts. The pooled standardized mean difference (SMD) was markedly positive, with the diamond-shaped point estimate and its entire 95% confidence interval (CI) positioned substantially to the right of the null effect line (*p* < 0.0001) (Figure 2). This statistically significant effect was consistently observed across the vast majority of the included studies, where individual SMDs frequently exceeded 2.0 (SMD = 3.15 (95% CI: 2.51–3.79), reinforcing the strong association between active PTB and increased systemic ADA activity.

However, the analysis revealed an extreme level of statistical heterogeneity, characterized by an *I*^2^ value of 95.6% and a highly significant Cochran’s Q test (*p* < 0.01). This degree of variability suggests substantial diversity in clinical populations, diagnostic thresholds, and laboratory methodologies utilized across the different study sites. Consequently, a random-effects model was employed to provide a more conservative and generalizable estimate of the effect size, acknowledging that the observed results represent a distribution of effects rather than a single true value. These findings demonstrate a strong association between elevated serum ADA levels and pulmonary tuberculosis; however, they do not establish diagnostic accuracy.

A sensitivity analysis excluding studies with potentially skewed data (defined as SD/mean ≥ 1) demonstrated that the pooled effect size remained materially unchanged, with no meaningful difference in magnitude or statistical significance compared to the primary analysis, indicating that the overall findings are robust to potential violations of the normality assumption.

#### 3.3.2. Subgroup Analysis by Geographic Region in PTB vs Healthy

**Figure 3:**
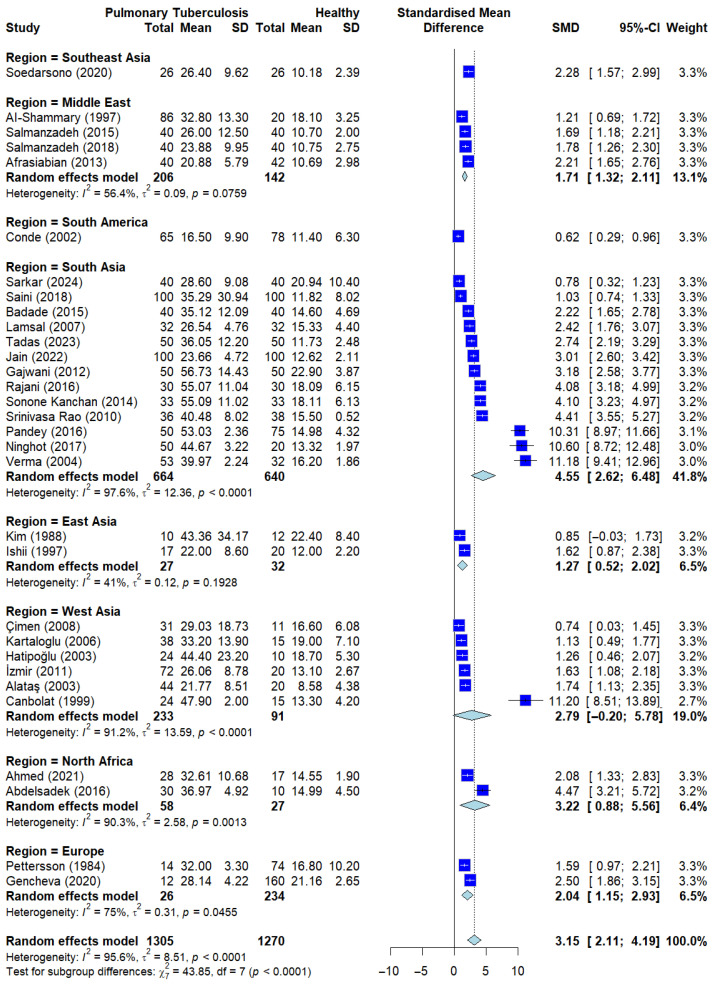
Subgroup analysis by geographical region. Analysis stratified by region/ethnicity showing consistent sADA elevation across all groups when compared to healthy controls. Deep blue squares represent individual study effect sizes, with horizontal lines indicating 95% confidence intervals. The size of each square reflects the study weight, and the blue diamond represents the pooled estimate from the random-effects model [13,14,15,16,17,20,21,22,23,25,26,27,28,29,30,31,32,33,34,35,36,37,38,39,40,41,42,43,44,45,46].

To investigate the consistency of the findings across diverse populations and identify potential sources of the substantial overall heterogeneity (*I*^2^ = 95.6), a subgroup analysis based on geographical regions was conducted (Figure 3). The analysis revealed that serum ADA levels were significantly and consistently higher in patients with pulmonary tuberculosis (PTB) compared to control groups across all identified territories.

The most consistent evidence was derived from South Asia and West Asia, which contributed the highest number of studies. Both regions demonstrated significant standardized mean differences (SMDs) with substantial intra-group heterogeneity of *I*^2^ = 97.6 and *I*^2^ = 91.2, respectively. Similarly, studies from South Africa showed point estimates significantly favoring the PTB group (*I*^2^ = 90.3). In regions represented by fewer studies, such as Southeast Asia and South America, the overall trend remained consistently positive, although intra-group heterogeneity was either not applicable due to single-study representation or varied.

Notably, lower levels of heterogeneity were observed in certain subgroups, specifically the Middle East (*I*^2^ = 56.4), East Asia (*I*^2^ = 41.0), and Europe (*I*^2^ = 71.0). The consistency of the positive SMD across these diverse ethnic and racial clusters, ranging from Caucasian to East Asian and African populations, suggests that serum ADA is consistently elevated across studies. These findings imply that the physiological activation of T-lymphocytes and subsequent ADA release in response to TB infection is a conserved immune mechanism regardless of ethnicity or environmental settings. However, as a high heterogeneity persisted within the majority of subgroups, these results indicate that geographical location and ethnic background are not the primary drivers of the variance in observed effect sizes, pointing instead toward potential variations in clinical severity or laboratory methodologies.

To further quantify the impact of geographical region as a categorical moderator, a mixed-effects meta-regression was conducted (Supplementary File S1). The results of the Test of Moderators indicated that geographical region did not significantly account for the heterogeneity observed between the studies (*QM* = 5.44, df = 7, *p* = 0.6064).

The model estimated the amount of residual heterogeneity (*I*^2^) at 98.89%, with a residual variance (*tau*^2^) of 9.15 (SE = 2.77). The *R*^2^ value of 0.00% further confirms that the geographical region has negligible explanatory power for the variance in serum ADA effect sizes. These findings imply that the high heterogeneity is likely driven by other factors not captured by regional categorization, such as variations in laboratory assay sensitivity.

#### 3.3.3. Subgroup Analysis by ADA Measuring Method in PTB vs Healthy

To investigate whether the technical approach to measuring enzyme activity influenced the study findings, a subgroup analysis was performed based on the laboratory methods used across the studies. The included studies were categorized into groups such as the Giusti-based colorimetric assay, automated biochemistry analyzers, and other enzymatic methods.

**Figure 4:**
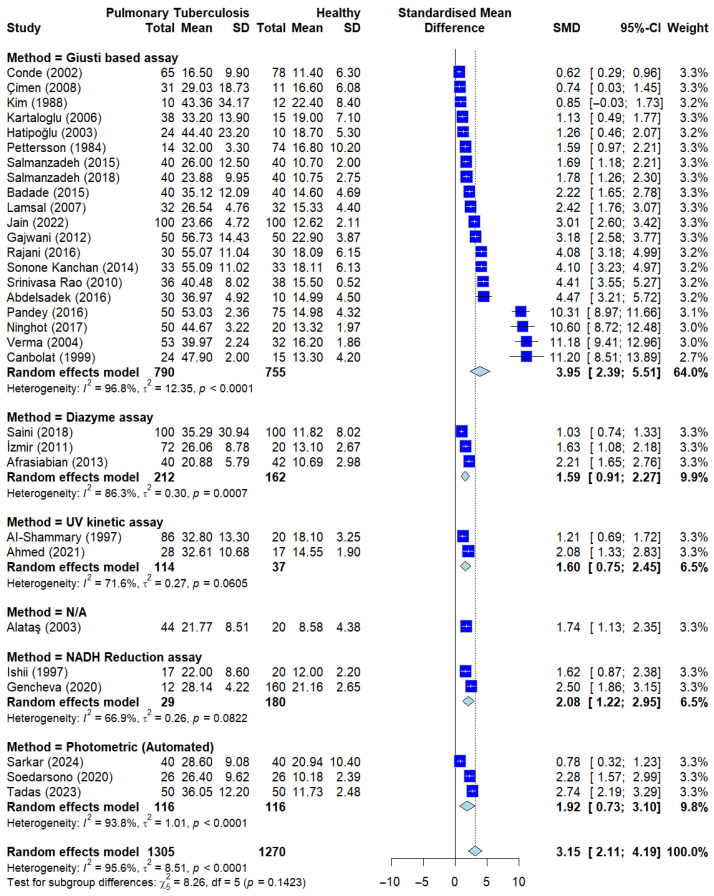
Subgroup analysis by sADA measuring method. Analysis stratified by different ADA methods showing consistent ADA elevation across all groups when compared to healthy controls. Deep blue squares represent individual study effect sizes, with horizontal lines indicating 95% confidence intervals. The size of each square reflects the study weight, and the blue diamond represents the pooled estimate from the random-effects model [13,14,15,16,17,20,21,22,23,25,26,27,28,29,30,31,32,33,34,35,36,37,38,39,40,41,42,43,44,45,46].

As illustrated in the Forest plot (Figure 4), serum ADA levels remained significantly higher in PTB patients than in healthy controls across all methodological subgroups. The Giusti-based assay, which remains a widely used manual method, showed a consistent and significant elevation in ADA activity, comparable to the results obtained from modern automated analyzers.

According to the quantitative meta-regression results provided in Supplementary File S1, the choice of ADA measurement method did not significantly contribute to the observed statistical heterogeneity. The Test of Moderators yielded a non-significant result (*QM* = 3.4313, df = 5, *p* = 0.6338), indicating that the variance in effect sizes across studies cannot be explained by the laboratory technique employed. Furthermore, the *R*^2^ value was calculated at 0.00%, confirming that the measurement method had negligible explanatory power for the residual heterogeneity (*I*^2^ = 98.88). These findings suggest that serum ADA is consistently elevated across studies, maintaining its statistical significance regardless of whether manual colorimetric techniques or automated high-throughput systems are utilized.

### 3.4. Pooled Standardized Mean Difference (SMD) of Serum ADA: PTB vs. Other Respiratory Diseases

#### 3.4.1. Pooled Standardized Mean Difference (SMD): PTB vs Other Diseases

The group-level differences in serum ADA levels were further evaluated by comparing patients with pulmonary tuberculosis (PTB) against those with other non-tuberculous respiratory diseases, such as pneumonia, lung cancer, and chronic obstructive pulmonary disease (COPD). This comparison is critical for assessing differences in ADA levels in clinically relevant comparison groups where clinicians must differentiate between various causes of pulmonary infiltrates.

**Figure 5:**
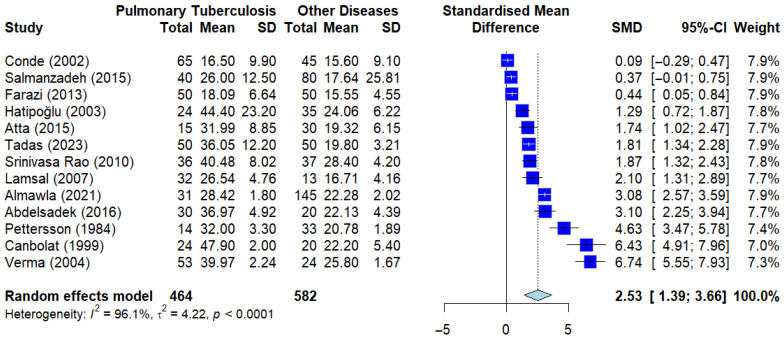
Forest plot of sADA levels in pulmonary tuberculosis (PTB) patients vs. other diseases. Deep blue squares represent individual study effect sizes, with horizontal lines indicating 95% confidence intervals. The size of each square reflects the study weight, and the blue diamond represents the pooled estimate from the random-effects model [13,18,19,21,23,24,27,33,36,39,44,45,46].

As illustrated in the Forest plot (Figure 5), the meta-analysis of the included studies demonstrated a significantly higher level of serum ADA in the PTB group compared to the OD group. The pooled standardized mean difference (SMD) was calculated at 2.06 (95% CI: 1.38–2.74, *p* < 0.0001), indicating a statistically significant elevation of the enzyme in tuberculous infections.

Despite the clear observed pattern, the analysis exhibited substantial statistical heterogeneity, with an *I*^2^ value of 98.15% and a residual variance (*tau*^2^) of 5.12. This high level of heterogeneity suggests that, while serum ADA is consistently higher in PTB patients, the magnitude of the difference varies across different study populations. These findings reinforce the potential of serum ADA as a biomarker showing consistent group-level differences, though its interpretation should consider the specific clinical context and potential causes of variance in different disease groups.

#### 3.4.2. Subgroup Analysis by Region of Serum ADA Levels in PTB vs Other Diseases

To evaluate whether group-level differences in serum ADA levels are influenced by geographical and ethnic factors, a subgroup analysis was performed by stratifying studies according to their geographical region (e.g., South Asia, Middle East, Africa, and others). This approach aims to account for potential variations in TB epidemiology, nutritional status, and genetic background that might impact systemic ADA levels.

**Figure 6:**
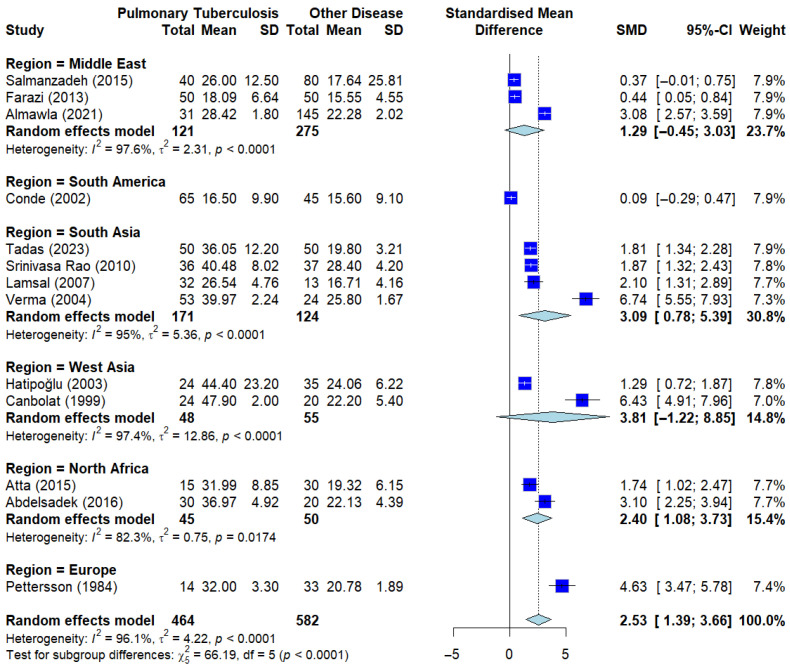
Subgroup analysis by geographical region. Analysis stratified by region/ethnicity showing consistent sADA elevation across all groups when compared to other diseases. Deep blue squares represent individual study effect sizes, with horizontal lines indicating 95% confidence intervals. The size of each square reflects the study weight, and the blue diamond represents the pooled estimate from the random-effects model [13,18,19,21,23,24,27,33,36,39,44,45,46].

The results, as visualized in the Forest plot (Figure 6), demonstrate that serum ADA levels remain significantly elevated in PTB patients compared to those with other respiratory diseases across all studied regions. While the overall trend remains consistent, the magnitude of the standardized mean difference (SMD = 2.53) shows some variation across regions, which likely contributes to the high global heterogeneity observed in this meta-analysis.

According to the meta-regression analysis performed to identify potential moderators (as detailed in Supplementary File S2), the Test of Moderators indicated that geographical region did not significantly account for the heterogeneity observed between the studies (*QM* = 3.56, df = 5, *p* = 0.5704). Furthermore, the model estimated the amount of residual heterogeneity (*I*^2^) at 98.15%, with a residual variance (*tau*^2^) of 4.78. The *R*^2^ value of 0.00% confirms that geographical location has negligible explanatory power regarding the variance in effect sizes. These findings suggest that serum ADA elevation in PTB is a broad, universal immunological phenomenon, and its biological association is not restricted to a specific geographical population.

#### 3.4.3. Subgroup Analysis by ADA Method of Serum ADA Levels in PTB vs. Other Diseases

To evaluate whether the laboratory technique used to measure adenosine deaminase activity influences the observed differences in ADA levels between pulmonary tuberculosis (PTB) and other respiratory diseases, we conducted a subgroup analysis based on the measurement methods. This analysis is important for evaluating the consistency of group-level differences in serum ADA across different laboratory methods.

**Figure 7:**
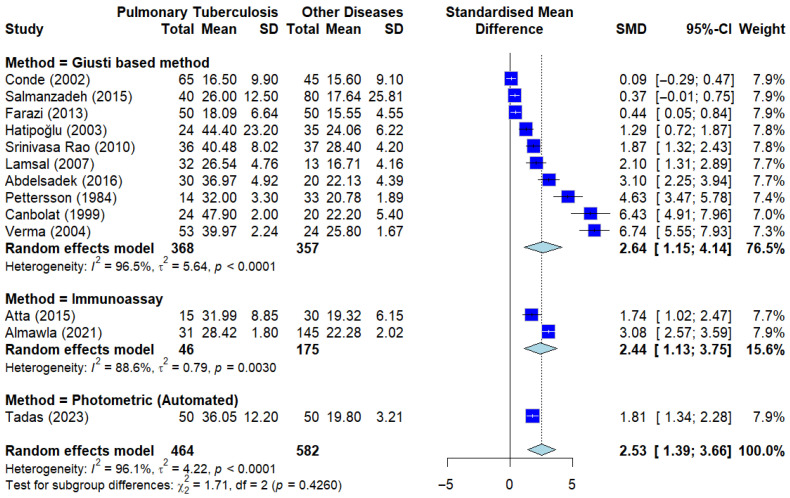
Subgroup analysis by sADA measuring method. Analysis stratified by different sADA methods showing consistent ADA elevation across all groups when compared to other diseases. Deep blue squares represent individual study effect sizes, with horizontal lines indicating 95% confidence intervals. The size of each square reflects the study weight, and the blue diamond represents the pooled estimate from the random-effects model [13,18,19,21,23,24,27,33,36,39,44,45,46].

As illustrated in the Forest plot (Figure 7), serum ADA levels remained consistently and significantly elevated in PTB patients compared to those with other pulmonary conditions across all methodological categories, including the traditional Giusti-based assay and various automated enzymatic analyzers.

The impact of the ADA measurement method as a categorical moderator was further examined, and the results are detailed in Supplementary File S2. The Test of Moderators indicated that the laboratory method did not significantly account for the heterogeneity observed between the studies (*QM* = 0.13, df = 2, *p* = 0.9386). Furthermore, the model estimated the amount of residual heterogeneity (*I^2^*) at 98.15%, with a residual variance (*tau^2^*) of 5.12 (SE = 2.37). The *R^2^* value of 0.00% confirms that the measurement method has negligible explanatory power for the variance in serum ADA effect sizes. These findings suggest that the elevation of serum ADA levels in PTB is a consistent, method-independent phenomenon, supporting a consistent direction of association across different measurement platforms, although the magnitude varied between studies.

## 4. Discussion

This meta-analysis synthesizes evidence from 34 independent studies and demonstrates that serum adenosine deaminase (ADA) levels are significantly elevated in patients with pulmonary tuberculosis (PTB) compared with both healthy individuals and patients with non-tuberculous respiratory diseases. The pooled effect sizes observed in the present analysis support the growing body of evidence suggesting that serum ADA is a biologically relevant marker associated with tuberculosis infection. Previous clinical studies consistently reported significantly higher serum ADA levels in patients with active TB compared with controls, reinforcing the potential role of ADA as a biomarker associated with disease activity [14,35,39]. Similar findings have been reported in several independent populations, including studies demonstrating markedly elevated ADA activity in PTB patients compared with individuals with other pulmonary conditions [24,38,47]. These observations collectively support the consistency in the direction of the association of serum ADA with pulmonary tuberculosis. Importantly, these findings should not be used to inform clinical decision-making, as they are derived from group-level comparisons rather than diagnostic accuracy measures.

These findings should be interpreted in the context of group-level differences rather than diagnostic accuracy. The standardized mean difference (SMD) quantifies the magnitude of separation between PTB and control groups, but does not provide clinically actionable measures such as sensitivity, specificity, likelihood ratios, or area under the receiver operating characteristic curve. Therefore, although serum ADA demonstrates a strong biological association with PTB, these results do not establish its standalone diagnostic performance. Future studies employing diagnostic test accuracy designs are necessary to determine their clinical applicability.

It is important to distinguish between the biological relevance and clinical applicability of serum ADA in pulmonary tuberculosis. Elevated ADA levels reflect the activation of cell-mediated immunity and increased T-lymphocyte activity during Mycobacterium tuberculosis infection, supporting its role as a biologically relevant marker of host immune response. However, biological association does not necessarily translate into clinical usefulness. In the absence of validated diagnostic thresholds and accuracy measures such as sensitivity and specificity, the ability of serum ADA to reliably distinguish PTB from other conditions in clinical practice remains uncertain. Therefore, while ADA reflects underlying disease-related immune activation, its role in clinical decision-making cannot be established based on the current evidence.

The biological basis for ADA elevation in tuberculosis is closely linked to the host’s cell-mediated immune response. ADA plays a critical role in purine metabolism and is essential for the proliferation and differentiation of activated T-lymphocytes, which are central to the immune defense against intracellular pathogens such as *M. tuberculosis*. During active infection, macrophages and T-cells accumulate at sites of infection and release ADA into the systemic circulation as part of the inflammatory response [35,48]. Elevated ADA activity, therefore, reflects the intensity of cellular immune activation and can serve as a biochemical indicator of host–pathogen interaction. Experimental and clinical investigations have shown that increased ADA activity correlates with immune activation and inflammatory burden in tuberculosis, further supporting its role as an immunological biomarker [7,49]. Additionally, several molecular and immunological studies have demonstrated that ADA-related pathways are involved in immune regulation and inflammatory signaling during infectious diseases, providing mechanistic support for its biological relevance [50].

A notable finding of the present meta-analysis is the consistent elevation of serum ADA across diverse geographical settings and laboratory methodologies. Previous studies have reported that ADA measurements remain reliable regardless of assay platform, including classical colorimetric methods such as the Giusti–Galanti assay and automated clinical chemistry analyzers [34,35]. Moreover, multiple clinical studies have demonstrated similar observed patterns in different populations, suggesting that ADA activity is relatively stable across ethnic and demographic groups [51,52]. This consistency supports the interpretation that serum ADA is associated with pulmonary tuberculosis; however, its ability to inform clinical diagnosis cannot be established based on the current evidence.

Despite the overall consistency in the direction of effect, substantial residual heterogeneity was observed (*I^2^* > 95%), indicating considerable variability across studies. This suggests that serum ADA levels are influenced by multiple clinical and methodological factors. Variations in patient characteristics, including disease severity, bacillary load, and host immune response, may contribute to differences in ADA levels. Importantly, several potential confounding factors may influence serum ADA activity, including HIV infection, coexisting inflammatory or infectious diseases, and malnutrition. These conditions may independently alter immune activation and lead to elevated ADA levels, thereby introducing confounding bias in the observed associations. Due to inconsistent reporting of these variables across the included studies, it was not feasible to perform subgroup analyses or meta-regression stratified by HIV status or other confounders. This limitation is particularly relevant in HIV-endemic settings, where immune dysregulation may substantially influence ADA levels. Therefore, the pooled estimates should be interpreted with caution, as the observed associations may be partially influenced by unmeasured confounding factors. Methodological differences, particularly the variability in reported ADA cut-off values across studies, are likely a major contributor to the observed heterogeneity. Several studies reported substantially different thresholds (e.g., 14 U/L [14], and 21 U/L [26]), reflecting differences in study populations, disease spectrum, and laboratory protocols. This wide variation limits comparability across studies and complicates clinical interpretation. From an implementation perspective, the absence of standardized and validated cut-off values represents a major barrier to the use of serum ADA in routine clinical practice. Without consistent thresholds, the interpretation of ADA levels remains context-dependent and may lead to inconsistent clinical decision-making.

Although serum ADA measurement is relatively simple and widely available, its clinical applicability cannot be established based on the current evidence. In the absence of validated diagnostic thresholds and accuracy measures, serum ADA should not be considered a diagnostic or screening tool in clinical practice. Previous clinical studies have reported elevated ADA levels in specific patient subgroups, such as smear-negative pulmonary tuberculosis. However, these findings are based on individual studies and are not supported by pooled diagnostic accuracy measures in the present analysis. Therefore, such observations should be interpreted cautiously, and further validation through diagnostic test accuracy studies is required [6,39]. Furthermore, several studies have suggested that ADA measurement has been explored in individual studies; however, such observations are not supported by the pooled diagnostic accuracy evidence in the present analysis, including sputum microscopy and molecular tests [19,53]. Importantly, these findings should not be used to inform clinical decision-making due to the substantial and largely unexplained heterogeneity observed in this meta-analysis. Rather, the results should be interpreted as hypothesis-generating, providing a basis for future studies designed to evaluate diagnostic accuracy.

Nevertheless, several limitations should be considered when interpreting these findings. First, the evidence of funnel plot asymmetry suggests the possibility of publication bias, which may result in an overestimation of the pooled effect size due to the preferential publication of studies reporting positive results. However, given the substantial heterogeneity observed (*I*^2^ > 95%), the interpretation of funnel plot asymmetry and Egger’s test should be approached with caution. In the presence of high heterogeneity, funnel plot asymmetry may arise from genuine between-study differences rather than true publication bias. Furthermore, statistical tests such as Egger’s regression are known to have reduced reliability under conditions of substantial heterogeneity and may produce misleading results. Therefore, the evidence for publication bias in this analysis should be interpreted cautiously. Second, although serum ADA levels were consistently elevated in patients with pulmonary tuberculosis, this finding reflects a biological association rather than diagnostic accuracy. Because ADA is involved in general T-cell activation, elevated levels can also occur in a variety of inflammatory or malignant conditions, including sarcoidosis, lymphoma, autoimmune disorders, and lung cancer [15,23]. Therefore, serum ADA should not be considered a standalone diagnostic test, and its clinical applicability remains uncertain without validation through diagnostic accuracy studies. Third, the high and largely unexplained heterogeneity limits the interpretability of pooled estimates, and the findings may not be directly generalizable to routine clinical practice. Fourth, this meta-analysis included only studies reporting mean and standard deviation values, thereby excluding studies that reported diagnostic accuracy measures such as sensitivity, specificity, or area under the curve. This represents a major methodological constraint, as it restricts the analysis to group-level differences and precludes the direct assessment of clinical diagnostic performance. Consequently, the findings reflect biological associations rather than clinically actionable evidence. A dual-analysis approach incorporating both continuous data and diagnostic test accuracy metrics would provide a more comprehensive evaluation; however, this was not feasible due to inconsistent reporting formats across studies. Finally, a substantial proportion of the included studies employed case–control designs, which are prone to spectrum bias. Such designs often compare well-defined disease and non-disease groups, potentially exaggerating differences in biomarker levels and leading to an overestimation of effect sizes. Therefore, the results should be interpreted as exploratory and hypothesis-generating rather than clinically definitive.

Future research should therefore focus on addressing these limitations through well-designed multicenter prospective studies. Large-scale studies are needed to establish standardized ADA cut-off values and to evaluate the influence of clinical variables such as disease severity, immune status, and comorbidities on ADA activity. Additionally, emerging biomarker research suggests that combining ADA with other inflammatory or molecular markers may significantly improve the ability to differentiate between PTB and non-TB conditions. Recent studies have proposed integrated biomarker panels incorporating ADA alongside systemic inflammatory indices and immune signaling molecules to enhance diagnostic precision and disease monitoring [7,49]. Such multi-marker strategies may represent a promising direction for the development of more accurate and clinically useful diagnostic tools for tuberculosis.

## 5. Conclusions

This study shows that serum ADA levels are significantly elevated in patients with pulmonary tuberculosis, supporting its role as a biologically relevant marker of immune activation. However, due to substantial heterogeneity and the absence of diagnostic accuracy measures (e.g., sensitivity and specificity), these findings should not be interpreted as evidence of clinical diagnostic utility. Importantly, these findings should not be used to inform clinical decision-making and should be interpreted as hypothesis-generating rather than clinically actionable. Further well-designed diagnostic test accuracy studies are required before serum ADA can be recommended for clinical application.

## Figures and Tables

**Table 1:** Characteristics and key highlights of included studies.

Author (Year)	[Ref]	Study Design	PTB (n)	Healthy, Other (n)	Serum ADA Measuring Method	Key Highlights
Abdelsadek (2016)	[13]	Prospective	30	10, 20	Giusti & Galanti	sADA showed higher percentage positivity (90%) compared to TT, ESR, and Radiography.
Afrasiabian (2013)	[14]	Cross-sectional	40	42, -	Diazyme Assay (Commercial Kit)	Reported a serum ADA value of 14 U/L in patients with pulmonary tuberculosis.
Ahmed (2021)	[15]	Cross-sectional	28	17, -	UV Kinetic (Semiautomated)	First study in Assiut evaluating serum ADA levels in patients with suspected pulmonary tuberculosis.
Al-Shammary (1997)	[16]	Retrospective	68	20, -	UV Kinetic (Ellis & Goldberg)	Assessed serum ADA levels in patients with negative AFB and PPD results.
Alatas (2003)	[17]	Prospective	44	20, -	N/A	Observed changes in serum ADA levels during treatment in pulmonary tuberculosis patients.
Almawla (2021)	[18]	Prospective	31	-, 145	ELISA	Compared ADA levels across four fluids; the highest levels were found in pleural fluid vs. serum.
Atta (2015)	[19]	Comparative	15	-, 30	ELISA	Reported higher ADA levels in sputum compared to serum in patients with pulmonary tuberculosis.
Badade (2015)	[20]	Prospective	40	40, -	Modified Giusti	Focused on challenges in identifying extrapulmonary and smear-negative pulmonary tuberculosis.
Canbolat (1999)	[21]	Comparative	24	15, 20	Giusti & Galanti	Noted PPD(+) healthy controls have significantly higher ADA than PPD(−) controls.
Cimen (2008)	[22]	Comparative	31	11, -	Giusti Method	Found ADA levels decreased as the number of resistant drugs increased in TB patients.
Conde (2002)	[23]	Prospective	65	78, 45	Giusti Method	Concluded ADA-2 isoform was not useful for differentiating PTB from other respiratory diseases in adults.
Farazi (2013)	[24]	Prospective	50	-, 50	Giusti Method	Reported differences in serum ADA levels compared with sputum microscopy in patients with pulmonary tuberculosis.
Gajwani (2012)	[25]	Comparative	50	50, -	Giusti & Galanti	Suggested combining ADA with serum ferroxidase: albumin ratio as a surrogate marker.
Gencheva (2020)	[26]	Comparative	12	160, -	NADH Reduction	Reported ADA levels greater than 21 U/L in patients with tuberculosis and inflammatory lung diseases.
Hatipoglu (2003)	[27]	Comparative	24	10, 35	Giusti Method	Reported an association between serum ADA levels and disease activity in suspected tuberculosis.
Ishii (1997)	[28]	Cohort	17	20, -	NADH Reduction (Commercial Kit)	Found ADA-2 activity correlated negatively with CD^3+^ and CD^4+^ lymphocyte counts.
İzmir (2011)	[29]	Prospective	72	20, -	Diazyme Assay	ADA levels correlated with the extent of radiological lesions and bacterial load.
Jain (2022)	[30]	Cross-sectional	100	100, -	Giusti & Galanti	Observed associations between serum ADA levels and disease status, as well as changes during treatment.
Kartaloglu (2006)	[31]	Prospective	38	15, -	Giusti Method	Observed ADA elevation in the 1st month of treatment, followed by a parallel decrease with recovery.
Kim (1988)	[32]	Observational	21	19, 8	Giusti Method	Used pleural fluid/serum ADA ratios to differentiate TB from malignancy.
Lamsal (2007)	[33]	Comparative	61	32, 13	Giusti & Galanti	Reported elevated ADA levels in patients where other clinical tests were inconclusive.
Ninghot (2017)	[34]	Case–Control	100	15, 35	Giusti Method (Commercial)	Reported elevated ADA levels in patients with extrapulmonary tuberculosis (e.g., meningitis, lymphadenitis).
Pandey (2016)	[35]	Cross-sectional	75	75, -	Modified Guisti	Reported a serum ADA value of 25 U/L in patients with pulmonary tuberculosis.
Pettersson (1984)	[36]	Prospective	14	74, 76	Modified Giusti	Indicated local synthesis of ADA within the pleural cavity.
Rajani (2016)	[37]	Comparative	60	30, 30	Giusti & Galanti	Found mean pleural fluid ADA significantly higher than serum ADA in TB patients.
Saini (2018)	[38]	Cohort	100	100, -	Diazyme Assay (Commercial Kit)	Confirmed fall in ADA levels after the intensive phase of treatment is a prognostic marker.
Salmanzadeh (2015)	[39]	Comparative	40	40, 40	Giusti & Galanti	Reported differences in serum ADA levels in patients from resource-limited settings.
Salmanzadeh (2018)	[40]	Cross-sectional	40	40, 40	Giusti & Galanti	Reported variation in ADA levels across different cutoff values in extrapulmonary tuberculosis.
Sarkar (2024)	[41]	Cohort	40	40, -	Fully Automated (Photometric)	Discovered a strong negative correlation between sADA levels and Ct values from CBNAAT
Soedarsono (2020)	[42]	Prospective	26	26, -	Fully Automated (Photometric)	Found ADA levels in AFB 3+ patients were the highest before and after treatment.
Sonone (2014)	[43]	Prospective	66	33, -	Giusti & Galanti	Reported elevated ADA levels in patients with pulmonary tuberculosis, with and without pleural effusion.
Srinivasa Rao (2010)	[44]	Comparative	128	45, 14	Giusti & Galanti	Sputum AFB-negative PTB cases showed elevated ADA at par with AFB-positive cases.
Tadas (2023)	[45]	Cross-sectional	100	50, 50	Fully Automated (Photometric)	Evaluated serum ADA levels for differentiating tuberculosis from other respiratory conditions.
Verma (2004)	[46]	Retrospective	67	35, 33	Giusti	Found ADA levels were significantly raised in PTB regardless of sputum AFB status.

ADA: adenosine deaminase, AFB: acid-fast bacilli, CBNAAT: Cartridge-Based Nucleic Acid Amplification Test, Ct: cycle threshold, ELISA: enzyme-linked immunosorbent assay, NADH: reduced form of Nicotinamide Adenine Dinucleotide, PTB: pulmonary tuberculosis, sADA: serum adenosine deaminase, TB: tuberculosis, N/A: not available. The statements in this table reflect findings reported in individual studies and should not be interpreted as evidence of diagnostic accuracy in the present meta-analysis, -: not reported or not included in the study.

## Data Availability

No new data were created or analyzed in this study.

## Supplementary Materials

The following supporting information can be downloaded at: https://www.mdpi.com/article/10.3390/ijerph23040498/s1, Supplementary Table S1: PRISMA 2020 checklist; Supplementary Table S2: Search strategies; Supplementary Table S3: Methodological quality assessment using the Newcastle–Ottawa Scale (NOS); Supplementary File S1: Funnel plot and linear regression of PTB vs healthy control; Supplementary File S2: Funnel plot and linear regression of PTB vs other disease control.

## Abbreviations

The following abbreviations are used in this manuscript:

ADA	Adenosine deaminase
sADA	Serum adenosine deaminase
ELISA	Enzyme-linked immunosorbent assays
SMD	Standardized mean difference
AFB	Acid-fast bacilli
Ct	Cycle threshold
EPTB	Extrapulmonary tuberculosis
NADH	Nicotinamide Adenine Dinucleotide
PTB	Pulmonary tuberculosis
CBNAAT	Cartridge-Based Nucleic Acid Amplification Test
