# Supplementary material for: Association of Serum ADA Levels in Pulmonary Tuberculosis: A Systematic Review and Meta-Analysis
Source: Int J Environ Res Public Health. 2026 Apr 14;23(4):498. doi: 10.3390/ijerph23040498 (PMC13115617; doi:10.3390/ijerph23040498)
Supplement: Supplementary file 1 [file ijerph-23-00498-s001.zip › Supplementary File S1_Linear regression test of funnel plot asymmetry.pdf]

Association of Serum ADA Levels in Pulmonary Tuberculosis: A Systematic Review and Meta-Analysis

Jirarat Songsri [1,2], Jongkonnee Thanasai [3], Jitbanjong Tangpong [1], Anchalee Chittamma [4] and Wiyada Kwanhian Klangbud [5,*]

School of Allied Health Sciences, Walailak University, Nakhon Si Thammarat 80160, Thailand; jirarat.so@wu.ac.th
[2] Faculty of Medicine, Mahasarakham University, Mahasarakham 44000, Thailand; jongkonnee@msu.ac.th
[3] Department of Pathology, Faculty of Medicine Ramathibodi Hospital, Mahidol University, Bangkok 10400, Thailand; anchalee.chi@mahidol.ac.th
[4] Medical Technology Program, Faculty of Science, Nakhon Phanom University, Nakhon Phanom 48000, Thailand; wiyadakwanhian@gmail.com
* Correspondence: wiyadakwanhian@gmail.com

Meta bias and Linear regression test of funnel plot asymmetry (PTB vs Healthy)

Linear regression test of funnel plot asymmetry

Test result: t = 5.91, df = 29, p-value < 0.0001
Bias estimate: 9.0506 (SE = 1.5327)

Details:
- multiplicative residual heterogeneity variance (tau^2 = 10.7945)
- predictor: standard error
- weight: inverse variance
- reference: Egger et al. (1997), BMJ

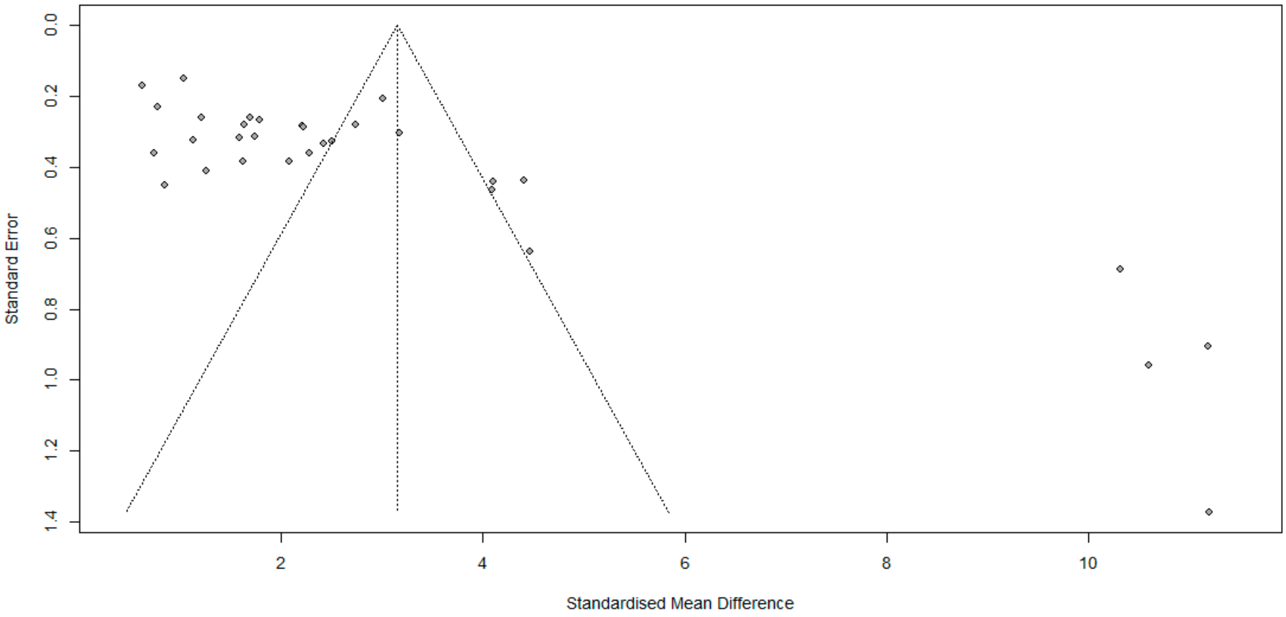

> m.reg <- metareg(m1, Region)

> print(m.reg)

Mixed-Effects Model (k = 31; tau^2 estimator: REML)

tau^2 (estimated amount of residual heterogeneity): 9.1499 (SE = 2.7682)

tau (square root of estimated tau^2 value): 3.0249

I^2 (residual heterogeneity / unaccounted variability): 98.89%

H^2 (unaccounted variability / sampling variability): 90.32

R^2 (amount of heterogeneity accounted for): 0.00%

Test for Residual Heterogeneity:

QE(df = 23) = 576.3688, p-val < .0001

Test of Moderators (coefficients 2:8):

QM(df = 7) = 5.4400, p-val = 0.6064

Model Results:

	estimate	se	zval	pval	ci.lb	ci.ub
intrcpt	1.2360	2.1593	0.5724	0.5671	-2.9963	5.4682
RegionEurope	0.8086	3.0479	0.2653	0.7908	-5.1652	6.7824
RegionMiddle East	0.4858	2.6397	0.1840	0.8540	-4.6881	5.6596
RegionNorth Africa	2.0190	3.0620	0.6594	0.5096	-3.9823	8.0204
RegionSouth America	-0.6123	3.7205	-0.1646	0.8693	-7.9044	6.6798
RegionSouth Asia	3.2878	2.3207	1.4168	0.1566	-1.2606	7.8362
RegionSoutheast Asia	1.0441	3.7341	0.2796	0.7798	-6.2746	8.3628
RegionWest Asia	1.4870	2.4999	0.5948	0.5519	-3.4127	6.3868

Signif. codes: 0 '***' 0.001 '**' 0.01 '*' 0.05 '.' 0.1 ' ' 1

```
> m.reg <- metareg(m1, Method)
> print(m.reg)

Mixed-Effects Model (k = 31; tau^2 estimator: REML)

tau^2 (estimated amount of residual heterogeneity):     9.2065 (SE = 2.6723)
tau (square root of estimated tau^2 value):             3.0342
I^2 (residual heterogeneity / unaccounted variability): 98.88%
H^2 (unaccounted variability / sampling variability):   89.44
R^2 (amount of heterogeneity accounted for):            0.00%

Test for Residual Heterogeneity:
QE(df = 25) = 645.0613, p-val < .0001

Test of Moderators (coefficients 2:6):
QM(df = 5) = 3.4313, p-val = 0.6338

Model Results:
                                   estimate      se    zval    pval
intrcpt                              1.6224  1.7575  0.9231  0.3559
```

MethodGiusti based assay	2.2949	1.8879	1.2156	0.2241
MethodN/A	0.1171	3.5204	0.0333	0.9735
MethodNADH Reduction assay	0.4394	2.7850	0.1578	0.8746
MethodPhotometric (Automated)	0.3088	2.4874	0.1241	0.9012
MethodUV kinetic assay	0.0172	2.7832	0.0062	0.9951

	ci.lb	ci.ub
intrcpt	-1.8223	5.0672
MethodGiusti based assay	-1.4053	5.9952
MethodN/A	-6.7828	7.0170
MethodNADH Reduction assay	-5.0191	5.8980
MethodPhotometric (Automated)	-4.5664	5.1840
MethodUV kinetic assay	-5.4377	5.4721

Signif. codes: 0 ‘***’ 0.001 ‘**’ 0.01 ‘*’ 0.05 ‘.’ 0.1 ‘ ’ 1
