# Supplementary material for: Association of Serum ADA Levels in Pulmonary Tuberculosis: A Systematic Review and Meta-Analysis
Source: Int J Environ Res Public Health. 2026 Apr 14;23(4):498. doi: 10.3390/ijerph23040498 (PMC13115617; doi:10.3390/ijerph23040498)
Supplement: Supplementary file 1 [file ijerph-23-00498-s001.zip › Supplementary File S2_Linear regression test of funnel plot asymmetry.docx (Other diseases and meta bias).pdf]

Association of Serum ADA Levels in Pulmonary Tuberculosis: A Systematic Review and Meta-Analysis

Jirarat Songsri [1,2], Jongkonnee Thanasai [3], Jitbanjong Tangpong [1], Anchalee Chittamma [4] and Wiyada Kwanhian Klangbud [5,*]

[1] School of Allied Health Sciences, Walailak University, Nakhon Si Thammarat 80160, Thailand; jirarat.so@wu.ac.th
[2] Faculty of Medicine, Mahasarakham University, Mahasarakham 44000, Thailand; jongkonnee@msu.ac.th
[3] Department of Pathology, Faculty of Medicine Ramathibodi Hospital, Mahidol University, Bangkok 10400, Thailand; anchalee.chi@mahidol.ac.th
[4] Medical Technology Program, Faculty of Science, Nakhon Phanom University, Nakhon Phanom 48000, Thailand; wiyadakwanhian@gmail.com
* Correspondence: wiyadakwanhian@gmail.com

Meta bias and Linear regression test of funnel plot asymmetry (PTB vs Other disease)

Test result: t = 5.42, df = 11, p-value = 0.0002
Bias estimate: 11.26 (SE = 2.08)

Details:
- multiplicative residual heterogeneity variance (tau^2 = 7.71)
- predictor: standard error
- weight: inverse variance
- reference: Egger et al. (1997), BMJ

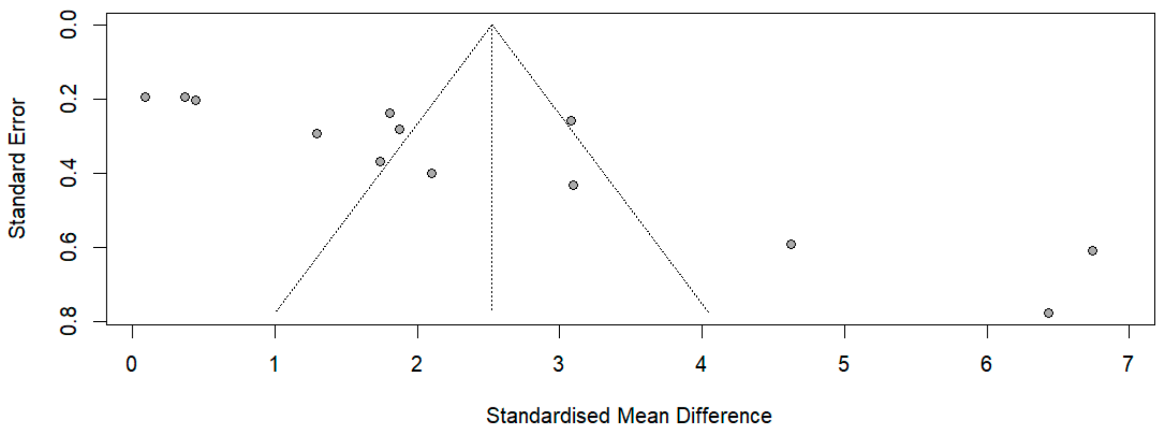

> m.reg <- metareg(m1, Region)

```
> print(m.reg)

Mixed-Effects Model (k = 13; tau^2 estimator: REML)

tau^2 (estimated amount of residual heterogeneity):     4.7802 (SE = 2.6376)
tau (square root of estimated tau^2 value):             2.1864
I^2 (residual heterogeneity / unaccounted variability): 98.15%
H^2 (unaccounted variability / sampling variability):   53.95
R^2 (amount of heterogeneity accounted for):            0.00%

Test for Residual Heterogeneity:
QE(df = 7) = 186.2703, p-val < .0001

Test of Moderators (coefficients 2:6):
QM(df = 5) = 3.8552, p-val = 0.5704

Model Results:
                     estimate      se     zval    pval     ci.lb
intrcpt                4.6263  2.2648   2.0427  0.0411    0.1874
RegionMiddle East     -3.3315  2.5959  -1.2833  0.1994   -8.4194
RegionNorth Africa    -2.2101  2.7569  -0.8017  0.4227   -7.6135
RegionSouth America   -4.5331  3.1539  -1.4373  0.1506  -10.7146
RegionSouth Asia      -1.5454  2.5230  -0.6125  0.5402   -6.4904
RegionWest Asia       -0.8919  2.7723  -0.3217  0.7477   -6.3255

                      ci.ub
intrcpt              9.0653  *
RegionMiddle East    1.7565
RegionNorth Africa   3.1932
RegionSouth America  1.6485
RegionSouth Asia     3.3995
RegionWest Asia      4.5417
---
Signif. codes:  0 ‘***’ 0.001 ‘**’ 0.01 ‘*’ 0.05 ‘.’ 0.1 ‘ ’ 1
```

```
> m.reg <- metareg(m1, Method)
> print(m.reg)

Mixed-Effects Model (k = 13; tau^2 estimator: REML)

tau^2 (estimated amount of residual heterogeneity):     5.1175 (SE = 2.3687)
tau (square root of estimated tau^2 value):             2.2622
I^2 (residual heterogeneity / unaccounted variability): 98.26%
H^2 (unaccounted variability / sampling variability):   57.42
R^2 (amount of heterogeneity accounted for):            0.00%

Test for Residual Heterogeneity:
QE(df = 10) = 263.9335, p-val < .0001

Test of Moderators (coefficients 2:3):
QM(df = 2) = 0.1266, p-val = 0.9386

Model Results:

                                 estimate     se     zval    pval
intrcpt                            2.6353  0.7285   3.6174  0.0003
MethodImmunoassay                 -0.2197  1.7721  -0.1240  0.9013
MethodPhotometric (Automated)     -0.8276  2.3886  -0.3465  0.7290

                                   ci.lb   ci.ub
intrcpt                           1.2075  4.0632  ***
MethodImmunoassay                -3.6929  3.2535
MethodPhotometric (Automated)    -5.5091  3.8539

---
Signif. codes:  0 ‘***’ 0.001 ‘**’ 0.01 ‘*’ 0.05 ‘.’ 0.1 ‘ ’ 1
```
