# Supplementary material for: Association of Serum ADA Levels in Pulmonary Tuberculosis: A Systematic Review and Meta-Analysis
Source: Int J Environ Res Public Health. 2026 Apr 14;23(4):498. doi: 10.3390/ijerph23040498 (PMC13115617; doi:10.3390/ijerph23040498)
Supplement: Supplementary file 1 [file ijerph-23-00498-s001.zip › Supplementary Table S2 _Search strategies.pdf]

Association of Serum ADA Levels in Pulmonary Tuberculosis: A Systematic Review and Meta-Analysis

Jirarat Songsri [1,2], Jongkonnee Thanasai [3], Jitbanjong Tangpong [1], Anchalee Chittamma [4] and Wiyada Kwanhian Klangbud [5,*]

[1] School of Allied Health Sciences, Walailak University, Nakhon Si Thammarat 80160, Thailand; jirarat.so@wu.ac.th
[2] Faculty of Medicine, Mahasarakham University, Mahasarakham 44000, Thailand; jongkonnee@msu.ac.th
[3] Department of Pathology, Faculty of Medicine Ramathibodi Hospital, Mahidol University, Bangkok 10400, Thailand; anchalee.chi@mahidol.ac.th
[4] Medical Technology Program, Faculty of Science, Nakhon Phanom University, Nakhon Phanom 48000, Thailand; wiyadakwanhian@gmail.com
* Correspondence: wiyadakwanhian@gmail.com

Supplementary Table S2. Search strategies form 3 databases

PubMed

Search number	Query	Search Details	Results	Date
1	("Tuberculosis, Pulmonary"[MeSH] OR "Pulmonary Tuberculosis" OR "Pulmonary TB" OR "Lung Tuberculosis" OR "Mycobacterium tuberculosis") AND ("Adenosine Deaminase"[MeSH] OR "Adenosine Deaminase" OR "ADA") AND ("Serum" OR "Blood" OR "Serum level*" OR "Serum concentration*")	("tuberculosis, pulmonary"[MeSH Terms] OR "Pulmonary Tuberculosis"[All Fields] OR "Pulmonary TB"[All Fields] OR "Lung Tuberculosis"[All Fields] OR "Mycobacterium tuberculosis"[All Fields]) AND ("Adenosine Deaminase"[MeSH Terms] OR "Adenosine Deaminase"[All Fields] OR "ADA"[All Fields]) AND ("Serum"[All Fields] OR "Blood"[All Fields] OR "serum level*"[All Fields] OR "serum concentration*"[All Fields])	168	23/2/2026

Embase

No.	Query	Results	Date

#1	('pulmonary tuberculosis'/exp OR 'pulmonary tuberculosis' OR 'pulmonary tb' OR 'lung tuberculosis') AND ('adenosine deaminase'/exp OR 'adenosine deaminase' OR 'ada') AND ('serum'/exp OR serum OR blood OR 'serum level' OR 'serum concentration')	277	23/Feb/26

Scopus

No.	Query	Results	Date
#1	(TITLE-ABS-KEY ("pulmonary tuberculosis" OR "pulmonary TB" OR "lung tuberculosis")) AND (TITLE-ABS-KEY ("adenosine deaminase" OR ADA)) AND (TITLE-ABS-KEY (serum OR blood OR "serum level" OR "serum concentration"))	219	23/Feb/26
