# Supplementary material for: Association of Serum ADA Levels in Pulmonary Tuberculosis: A Systematic Review and Meta-Analysis
Source: Int J Environ Res Public Health. 2026 Apr 14;23(4):498. doi: 10.3390/ijerph23040498 (PMC13115617; doi:10.3390/ijerph23040498)
Supplement: Supplementary file 1 [file ijerph-23-00498-s001.zip › Supplementary Table S3 _NOS.pdf]

Association of Serum ADA Levels in Pulmonary Tuberculosis: A Systematic Review and Meta-Analysis

Jirarat Songsri [1,2], Jongkonnee Thanasai [3], Jitbanjong Tangpong [1], Anchalee Chittamma [4] and Wiyada Kwanhian Klangbud [5,*]

[1] School of Allied Health Sciences, Walailak University, Nakhon Si Thammarat 80160, Thailand; jirarat.so@wu.ac.th
[2] Faculty of Medicine, Mahasarakham University, Mahasarakham 44000, Thailand; jongkonnee@msu.ac.th
[3] Department of Pathology, Faculty of Medicine Ramathibodi Hospital, Mahidol University, Bangkok 10400, Thailand; anchalee.chi@mahidol.ac.th
[4] Medical Technology Program, Faculty of Science, Nakhon Phanom University, Nakhon Phanom 48000, Thailand; wiyadakwanhian@gmail.com
* Correspondence: wiyadakwanhian@gmail.com

Supplementary Table S3. Methodological Quality Assessment Using the Newcastle-Ottawa Scale (NOS). Quality appraisal of the included studies based on three domains: selection of study groups, comparability of cases and controls (specifically age and sex matching), and ascertainment of exposure/outcome. Studies were categorized as High (7–9 stars), Moderate (4–6 stars), or Low (0–3 stars) quality.

Study (Author, Year)	[Ref]	Selection (Group Definition)	Comparability (Age/Sex Matching)	Outcome/Exposure (Methodology)	Overall Quality
Abdelsadek (2016)	[13]	Adequate: Prospective; culture confirmation.	Inadequate: Significant age difference (p<0.01).	Standardized: Guisti and Galanti method.	Moderate
Afrasiabian (2013)	[14]	Adequate: Smear-positive cases hospitalized.	Inadequate: Notable age gap (59 vs 49 yrs).	Standardized: Diazyme ADA kit.	Moderate
Ahmed (2021)	[15]	Adequate: AFB/Culture confirmed PTB/EPTB.	Inadequate: Authors noted lack of age matching.	Standardized: Semiautomated GLDH/UV kinetic.	Moderate
AI-Shammary (1997)	[16]	Adequate: Retrospective; culture confirmed.	Fair: No detailed demographic matching data provided.	Standardized: Kinetic method of Ellis and Goldberg.	Moderate
Alatas (2003)	[17]	Adequate: Role in diagnosis and follow-up.	Fair: Well-defined groups but matching details limited.	Standardized: Measurement not detailed in abstract.	Moderate
Almawla (2021)	[18]	Adequate: Real-Time PCR confirmation.	Adequate: No significant age difference (p=0.933).	Standardized: ELISA technique.	High
Atta (2015)	[19]	Inadequate: Very small sample size (n=15).	Inadequate: Significant age mismatch between groups.	Standardized: Specific immunoassay (Gusti method).	Low

Study (Author, Year)	[Ref]	Selection (Group Definition)	Comparability (Age/Sex Matching)	Outcome/Exposure (Methodology)	Overall Quality
Badade (2015)	[20]	Adequate: Prospective; newly diagnosed cases.	Adequate: Comparable control group criteria.	Standardized: Modified Giusti method.	High
Canbolat (1999)	[21]	Adequate: All cases culture-positive.	Adequate: Comparable age distribution (means ~37-40).	Standardized: Galanti and Giusti method.	High
Cimen (2008)	[22]	Adequate: Classified via WHO drug resistance criteria.	Inadequate: Statistically important age difference.	Standardized: Giusti method.	Moderate
Conde (2002)	[23]	Adequate: Prospective; gold standard culture.	Inadequate: Controls significantly younger (p=0.001).	Standardized: Giusti's method.	Moderate
Farazi (2013)	[24]	Adequate: Prospective; 4 well-defined groups.	Adequate: No significant differences in age/sex/residence.	Standardized: Giusti method.	High
Gajwani (2012)	[25]	Adequate: Newly diagnosed sputum-positive cases.	Adequate: Fifty age and sex matched healthy controls.	Standardized: Giusti and Galanti principle.	High
Gencheva (2020)	[26]	Adequate: 66 patients measured.	Fair: Used previous study results for controls.	Standardized: Spectrophotometric NADH reduction.	Moderate
Hatipoglu (2003)	[27]	Adequate: Culture and biopsy confirmation.	Inadequate: Significant age mismatch (63 vs 21 yrs).	Standardized: Guisti's colorimetric method.	Moderate
Ishii (1997)	[28]	Inadequate: Very small sample size (n=17).	Inadequate: Limited detailed demographic control data.	Standardized: Colorimetric kit (Giusti principle).	Low
İzmir (2011)	[29]	Adequate: Culture-confirmed cases followed.	Adequate: Included comparative data for healthy group.	Standardized: Calometric method (Diazyme).	High
Jain (2022)	[30]	Adequate: Microbiology-confirmed cases.	Adequate: Age and sex matched (44.06 vs 44.93 yrs).	Standardized: Giusti & Galanti principle.	High
Kartaloglu (2006)	[31]	Adequate: Prospective follow-up of smear+ cases.	Adequate: Healthy and age-matched men.	Standardized: Giusti's method.	High
Kim (1988)	[32]	Adequate: 37 cases; cytologic/histologic confirmation.	Fair: Control group was 12 healthy adults.	Standardized: Colorimetric assay of Giusti.	Moderate
Lamsal (2007)	[33]	Adequate: Defined by treatment response or smear.	Adequate: Healthy subjects from same geographical area.	Standardized: Guisti and Gallanti method.	High

Study (Author, Year)	[Ref]	Selection (Group Definition)	Comparability (Age/Sex Matching)	Outcome/Exposure (Methodology)	Overall Quality
Ninghot (2017)	[34]	Adequate: Confirmed by smear, culture, cytology.	Adequate: No significant differences in age/sex.	Standardized: Commercial Giusti kit.	High
Pandey (2016)	[35]	Adequate: Clinical and radiological confirmation.	Inadequate: Controls were significantly younger.	Standardized: Modified Guisti and Galanti method.	Moderate
Pettersson (1984)	[36]	Adequate: Culture or biopsy confirmation.	Adequate: Controls were 74 healthy hospital personnel.	Standardized: Modified Giusti method.	High
Rajani (2016)	[37]	Adequate: 120 subjects; 4 categories.	Adequate: Controlled age group (25-75 yrs).	Standardized: Giusti and Galanti method.	High
Saini (2018)	[38]	Adequate: Cohort study; smear +/- cases.	Adequate: 3 groups matched for age and gender.	Standardized: Photometric enzymatic assay.	High
Salmanzadeh (2015)	[39]	Adequate: PTB diagnosed via NTP protocol.	Adequate: 160 sex and age-matched subjects.	Standardized: Giusti and Galanti calorimetry.	High
Salmanzadeh (2018)	[40]	Adequate: Smear and culture confirmed EPTB.	Inadequate: Age disagreement between cancer/TB groups.	Standardized: Galanti and Guisti method.	Moderate
Sarkar (2024)	[41]	Adequate: Presumptive cases via ZN/CBNAAT.	Adequate: Age and sex-matched healthy controls.	Standardized: Automated analyzer.	High
Soedarsono (2020)	[42]	Adequate: New cases with positive rapid molecular tests.	Adequate: Age/sex comparable (42.8 vs 39.6 yrs).	Standardized: Enzymatic colorimetric method.	High
Sonone (2014)	[43]	Adequate: Prospective; 132 subjects.	Adequate: Age and sex matching performed.	Standardized: Guisti and Galanti method.	High
Srinivasa Rao (2010)	[44]	Adequate: 142 suspected cases evaluated.	Adequate: No significant difference in age/sex groups.	Standardized: Sensitive colorimetric method.	High
Tadas (2023)	[45]	Adequate: 200 randomly assigned subjects.	Adequate: Age & sex matched groups.	Standardized: Fully automated Biochemistry Analyzer.	High
Verma (2004)	[46]	Adequate: 100 patients; 5 subgroups.	Adequate: No significant differences in age/sex groups.	Standardized: Sensitive colorimetric method.	High

NOS: Newcastle-Ottawa Scale (The tool used for quality assessment), PTB: Pulmonary Tuberculosis, EPTB: Extra-pulmonary Tuberculosis, AFB: Acid-Fast Bacilli, CBNAAT: Cartridge-Based Nucleic Acid Amplification Test, ZN: Ziehl-Neelsen, PCR: Polymerase Chain Reaction, ELISA: Enzyme-Linked Immunosorbent Assay, GLDH/UV: Glutamate Dehydrogenase / Ultraviolet (Kinetic method for ADA), NTP: National Tuberculosis Program
